# Combined Endoscopic and Percutaneous Retrieval of a Retained 4-Wire Ureteral Stone Basket

**DOI:** 10.1089/cren.2015.29008.agk

**Published:** 2015-10-01

**Authors:** Adam G. Kaplan, Glenn M. Preminger, Michael E. Lipkin

**Affiliations:** Division of Urologic Surgery, Duke University Medical Center, Durham, North Carolina.

## Abstract

Complex endourologic procedures may require the use of a combined ureteroscopic and percutaneous approach. Endoscopic removal of a retained 4-wire ureteral stone basket is particularly complex, as broken tines can potentially injure the ureter if the basket is removed in a retrograde manner. The patient in this case presented with a ureteral stone basket embedded within the urothelium of the upper pole of the kidney. Holmium laser incision of the overlying urothelium allowed retrieval of the basket, although the tines were broken. Endoscopically guided percutaneous access to the kidney was obtained to allow for direct passage of the retained basket out of a nephrostomy sheath, thereby protecting the kidney.

## Clinical History

**FIG. 1.**
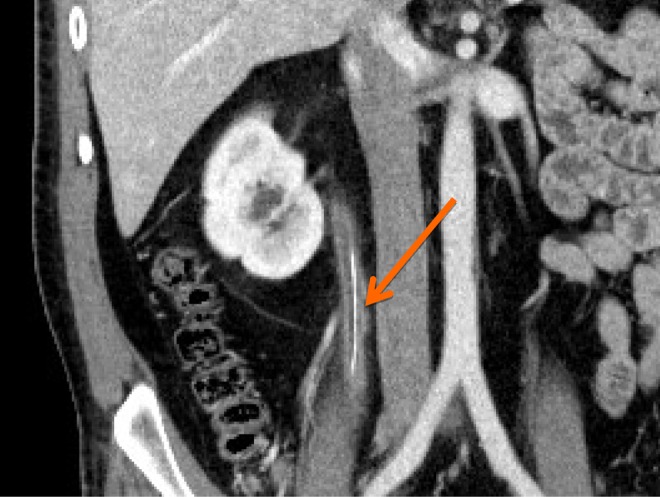
Retained 4-wire ureteral stone basket seen as a linear structure within the ureter.

A 40-year-old man with a history of recurrent nephrolithiasis, having undergone ureteroscopy in the past, presented to the emergency room with severe right-sided renal colic. He was found on CT to have a right mid-ureteral stone and foreign body in the proximal ureter extending to the upper pole in the kidney (Fig. 1). He was without fevers or leukocytosis and his urine showed no signs of significant infection. His serum creatinine was normal and his urine culture was negative. He was otherwise without medical problems and had a history of ureteroscopy 3 years ago at an outside institution. He took no medications and had no allergies. He was a 15 pack year smoker, but no alcohol or drug use. Family history was negative for stone disease.

## Physical Examination

The patient had normal vital signs. He appeared healthy but in moderate distress from pain. His physical examination was unremarkable, and he had no significant flank or abdominal tenderness.

## Diagnosis

Initial ureteroscopy was performed to clear the ureteral stone and the foreign body could then be visualized. A 10 cm segment of a 4-wire basket was found in the upper ureter extending proximally to the upper pole, where it was encased under a layer of urothelium. Stone had formed over the exposed wires. The foreign body was presumably left over from his previous ureteroscopy 3 years ago.

## Intervention

**FIG. 2.**
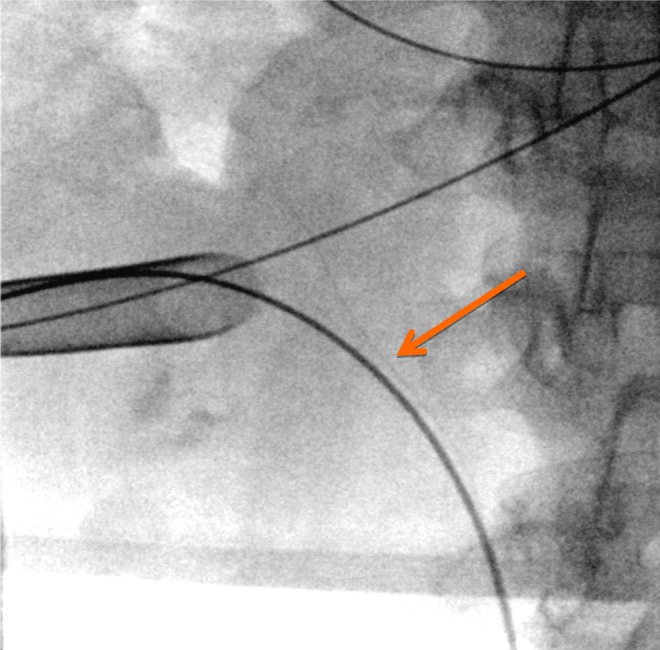
Retained 4-wire basket (*orange arrow*) shown with percutaneous access sheath in position.

**FIG. 3.**
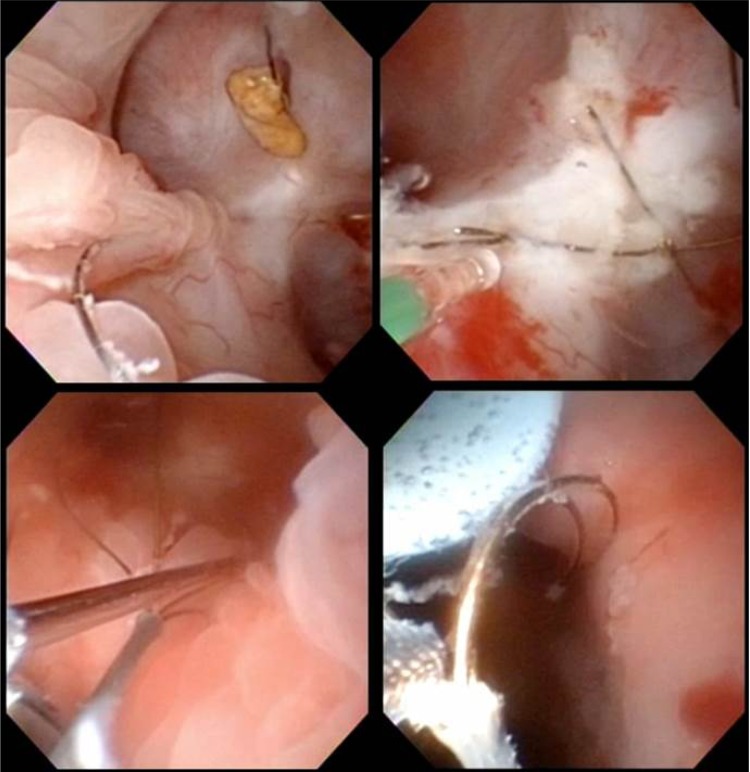
Clockwise from *top left*: Ingrown 4-wire basket with overlying urothelium and stone; holmium laser incision of the urothelium overlying the basket; grasping the unroofed basket with Triclaw 3-wire grasper; passing the basket antegrade through the ureteral access sheath.

The patient was positioned in the prone split-leg position. Retrograde access was obtained and an access sheath was placed. The ureteroscope was passed into the upper pole of the right kidney and the 4-wire basket was identified. A 200 mm holmium laser fiber at settings of 1.0 J and 10 Hz was used to fragment the stones and incise the overlying urothelium, thereby freeing the basket. The basket tines were severed at two locations making the removal of the basket through the ureteral access sheath impossible. An interpolar posterior calyx was chosen for the placement of a percutaneous access sheath, which was placed under fluoroscopic and direct endoscopic visualization (Fig. 2). The basket was then grabbed with a Triclaw 2.4F 3-wire grasper (UroGyn Medical, Inc., Valapraiso, IN) and passed retrograde through the percutaneous access sheathsheath (Fig. 3). Additional stone fragments were grasped and removed in the same manner. A Double-J 26 cm ×6F ureteral stent was left postoperatively and the access sheath was removed.

## Follow-Up

The patient recovered well from surgery and was discharged on postoperative day 1. His stent was removed on postoperative day 6, and he remains stone free and without hydronephrosis at his 3-month follow-up based on renal ultrasonography and kidney, ureter, and bladder radiograph (KUB)/digital tomography. Stone analysis revealed 50% Ca oxalate monohydrate, 20% calcium oxalate dehydrate, and 30% calcium phosphate (apatite).

As per our routine at Duke, we performed a renal ultrasound and KUB/digital tomogram on follow-up, which confirmed stone-free and foreign body-free status.

## Outcomes

A combined endoscopic and percutaneous approach is feasible and appropriate for complex endoscopic cases^1^ and has been used for the removal of severely encrusted stents and other endourologic challenges with success.^2^ Retrieval of a retained foreign body, such as a cut portion of a 4-wire basket, can be approached in a similar manner to avoid potential damage to the ureter.

## Abbreviations Used

CT: computed tomography
KUB: kidney, ureter, and bladder radiograph
